# A nationwide mobility service guarantee for Austria: possible design scenarios and implications

**DOI:** 10.1186/s12544-022-00550-5

**Published:** 2022-06-20

**Authors:** Barbara Laa, Takeru Shibayama, Tadej Brezina, Stefan Schönfelder, Dragana Damjanovic, Elke Szalai, Manuel Hammel

**Affiliations:** 1 Institute of Transportation, TU Wien, Karlsplatz 13/230-01, 1040 Vienna, Austria; 2 Institute for Transport and Logistics Management, WU Vienna, Vienna, Austria; 3 Research Unit Legal Sciences, TU Wien, Vienna, Austria; 4 Planung&Vielfalt, Wiener Neustadt, Austria

**Keywords:** Sustainable transport, Transport behaviour, Public transport, Mobility as a service

## Abstract

**Background:**

We present a sketch for a nationwide “Mobility Service Guarantee” (MSG) for Austria. The approach follows a new paradigm, turning away from car-centric transport policy and planning and towards the extensive provision of public transport. The approach is complemented by the provision of demand-responsive transport services and the support of vehicle sharing as well as active mobility. This combination should serve as an effective alternative option to the use of private cars.

**Purpose:**

The aim of this study is to develop a tangible definition of a nationwide MSG in the Austrian context as well as possible design scenarios.

**Methods:**

We took a multi-dimensional approach, using literature review to research existing concepts of mobility guarantees, analysing secondary data on current mobility behaviour and public transport provision and conducting stakeholder workshops.

**Results:**

We define possible scenarios envisioning a nationwide MSG including different extents of maximum distance to public transport stops and minimum frequency. We discuss the limitations of the MSG with respect to spatial, temporal and modal coverage, as well as how such a guarantee could be embedded in the existing legal system.

**Conclusions:**

We conclude that a nationwide MSG could be an important element of sustainable transport policy that should be embedded in a wider strategy of infrastructure and service design as well as demand management.

## Introduction

Transport systems focusing on the private car are increasingly proving to be problematic. This is not only due to environmental impacts but also considering social justice issues. The largest part of transport CO_2_ emissions in the transport sector are attributed to passenger cars [22] In Austria, CO_2_ emissions from transport have increased by 74% between 1990 and 2019 [44]. Since 2014, emissions have risen continuously until 2020, while there was a reduction by 14% (− 3.4 Mio. t CO_2_e) [2] due to decreased traffic during the COVID-19 pandemic.

As simulations have shown [8, 17], technological solutions—such as a shift to electric vehicles—alone will not be capable of reaching climate targets. Instead, far-reaching transformations to shift the transport system away from the private car are necessary.

Environmental challenges are also connected to questions of social justice, which demands solutions to tackle social and environmental challenges alike [33]. Car-oriented transport planning and policy lead to car dependence [36] which is linked to transport poverty [34]. Pursuing the current car-centric practices will only create further lock-in effects that make it more difficult to achieve a modal shift and to enable accessibility for people without access to private cars [9, 12, 15, 32, 35, 47].

In response to these challenges, in 2020 the Austrian federal government manifested to create a nationwide “Mobility Service Guarantee” (MSG) into its national political agenda [7]. Not yet translated into a workable strategy, the MSG is conceptualised as an approach to promote sustainable mobility as well as to improve mobility for persons without access to an own car. As such, the MSG stands on the provision of high-quality public transport (PT) services, complemented by demand responsive transport (DRT) services as well as “smart mobility services” (such as shared vehicles and car-pooling) where traditional PT cannot be delivered efficiently. In addition, booking, payment and usage need to be as easy and integrated as possible (c.f. a Mobility as a Service approach (MaaS) [14, 23]). All this together is expected to offer a high degree of service availability and reliability for travellers using or willing-to-use public transport and an attractive alternative to the private car.

This MSG approach is understood in a broader context of a new goal-oriented planning paradigm in contrast to “traditional” transport planning. The latter has been for long based solely on the provision of (car) infrastructure to fulfil predicted growth of (car) traffic (“predict and provide”) [16, 37]. In goal-oriented planning, a desired normative future state is set as an objective, and measures that are necessary to reach this desired state are planned in a backward manner. In the European urban context, the uptake of Sustainable Urban Mobility Plans [39, 45] accelerated the diffusion of goal-oriented planning in urban areas, and evidence about its efficiency is gradually cumulating (e.g. [41]. Meanwhile in the context of rural areas and on the nationwide scale, it still remains relatively novel. The first Austrian national transport master plan to include measurable targets (including the reduction of CO2 emissions) was published in 2013 [10]. The latest national mobility master plan [5] is the first one to include a path showing the necessary reduction of vehicle-kilometres travelled by private cars in line with the goal of climate neutrality in 2040.

The aim of this study is to develop a tangible definition of a nationwide MSG in this Austrian context as well as to develop possible design scenarios. To address this, we took a multi-dimensional approach with the following methods: (1) Literature review on existing concepts of mobility guarantees, (Sect. 2), (2) Analysis of secondary data on mobility behaviour and status quo of public transport provision in Austria (Sect. 3) (for a detailed GIS-based analysis please refer to Brezina and Hammel [6], p. 122 ff. and (3) Stakeholder workshops using the holistic pattern mining method (Sect. 4). Together with the analysis of existing legal framework, the results were synthesized to make a framework definition (Sect. 5). This framework definition is translated to possible scenarios of guarantee (Sect. 6).

At large, we aim to serve this study as a starting point to discuss the potential role and composition of such a guarantee. This research could be understood as a kind of gedankenexperiment (thought experiment) to form a basis towards MSG.

## Literature review

Different approaches of “mobility guarantees” or similar concepts exist. For example, the “Guaranteed Ride Home” programs in the U.S. that ensure a financial compensation in case a commute return trip cannot be made using car-pooling or PT [27]. A similar French approach covering car-pooling is known as the “Covoiturage Régulier” (regular car-pooling) [1, 31] and various German provinces and transport authorities provide financial compensation for delayed or cancelled PT services [24].

There are a few experimental approaches using the term “mobility guarantee” that go beyond compensation for cancelled trips only. One is the German research project “Mobilfalt” (started in 2013) that aims at supplementing existing PT services in a rural area by organising private and commercial ride-sharing and ride-hailing. This is expected to bridge spatial and temporal gaps in PT availability and reduce car dependency [38, 43]. The German project “Garantiert mobil im Odenwaldkreis” (guaranteed mobile in Odenwaldkreis) with similar objectives is in operation since 2017 [25].

Far reaching approaches are the mobility acts in France (LOM LOI n° 2019–1428) and in Berlin (MobG BE, GVBl. 2018). These national and regional laws show certain aspects of a comprehensive “mobility guarantee” and the promotion of public transport and active mobility. However, they mainly set objectives, while the concretisation and implementation remain the responsibility of the administration applying existing planning instruments with the given political and budgetary constraints.

A different approach is to set quality standards for PT especially for rural areas where PT coverage is often fragmented. In a study for the German ADAC [13], suggestions are made for concrete minimum standards in PT provision: for towns with more than 500 inhabitants, there should be a 1-h interval between 6 a.m. and 10 p.m.; trips with PT should take no more than 30% longer than with private car and at least 80% of inhabitants should live within 300 m of a PT stop. Herget et al. [19] support the suggestions but point out that the legal basis and financial feasibility for this approach are still missing. They further conclude that reaching these standards would require a focus on DRT services in rural areas.

The idea of implementing a nationwide MSG to enable mobility without the need to use a private car is relatively new; especially as a tool for sustainability transition in the transport sector as well as for social inclusion, to enable mobility for everyone, including those who are not able to or cannot afford to use a private car. Therefore, in the research presented here, we aim to find a shared understanding of how such a nationwide MSG could and should look like and design different implementation scenarios.

## Starting point towards MSG: current travel behaviour and mobility services in Austria

### Travel behaviour

The travel behaviour in Austria shows a distinct regional heterogeneity between the capital city Vienna and the rest of the country. The average modal share in Austria for weekdays shows 17% walking, 7% cycling, 59% car, and 17% PT of all trips [11]. Vienna shows a much higher modal share in public transport use and low share of car trips. PT usage in Vienna was high and stable at around 38%–40% between 2012 and 2019, with an exception of drop to 27% in 2020 due to COVID-19 [29], Wiener [46]. The share of car trips has remained stable at around 27% since 2015 [29], Wiener [46].

According to the most recent national travel survey [11], around 80% of Austrian households own a car while only 21% of persons over 15 years old own a monthly or annual pass for PT. With more than 50%, the ownership rate in Vienna is much higher than the national average. The temporal distribution of trips shows a distinctive day-night pattern with significantly less mobility outside the period between 5 a.m. and about 10 p.m. [11].

### Mobility services

While a variety of new mobility services such as car and bike sharing [42] are provided in a limited area of Austria such as Vienna and some large cities, public transport serves as the primary mobility service on a nationwide scale.

Brezina and Hammel [6] conducted an in-depth GIS-analysis of PT coverage in Austria. They used the “Public Transport Service Quality Level” (PTSQL) classification defined by ÖROK (Austrian Conference on Spatial Planning,an organisation coordinating spatial development at the national level) for their analysis [20]. PTSQL is a spatio–temporal categorization of areas into classes A to G, with class A being the highest rank with very good PT services (usually in large cities) and class G being the lowest, representing only very basic services in rural areas. The minimum requirement for class G is a bus station with at least four departures per direction per day (i.e., interval of less than 210 min.) in a maximum distance of 500 m or a train station with hourly interval in a maximum distance of 1,250 m. In this research, areas with the classes A-G are classified with the area with PT services available while areas outside of the classes A-G are classified as no PT services available.

**Fig. 1:**
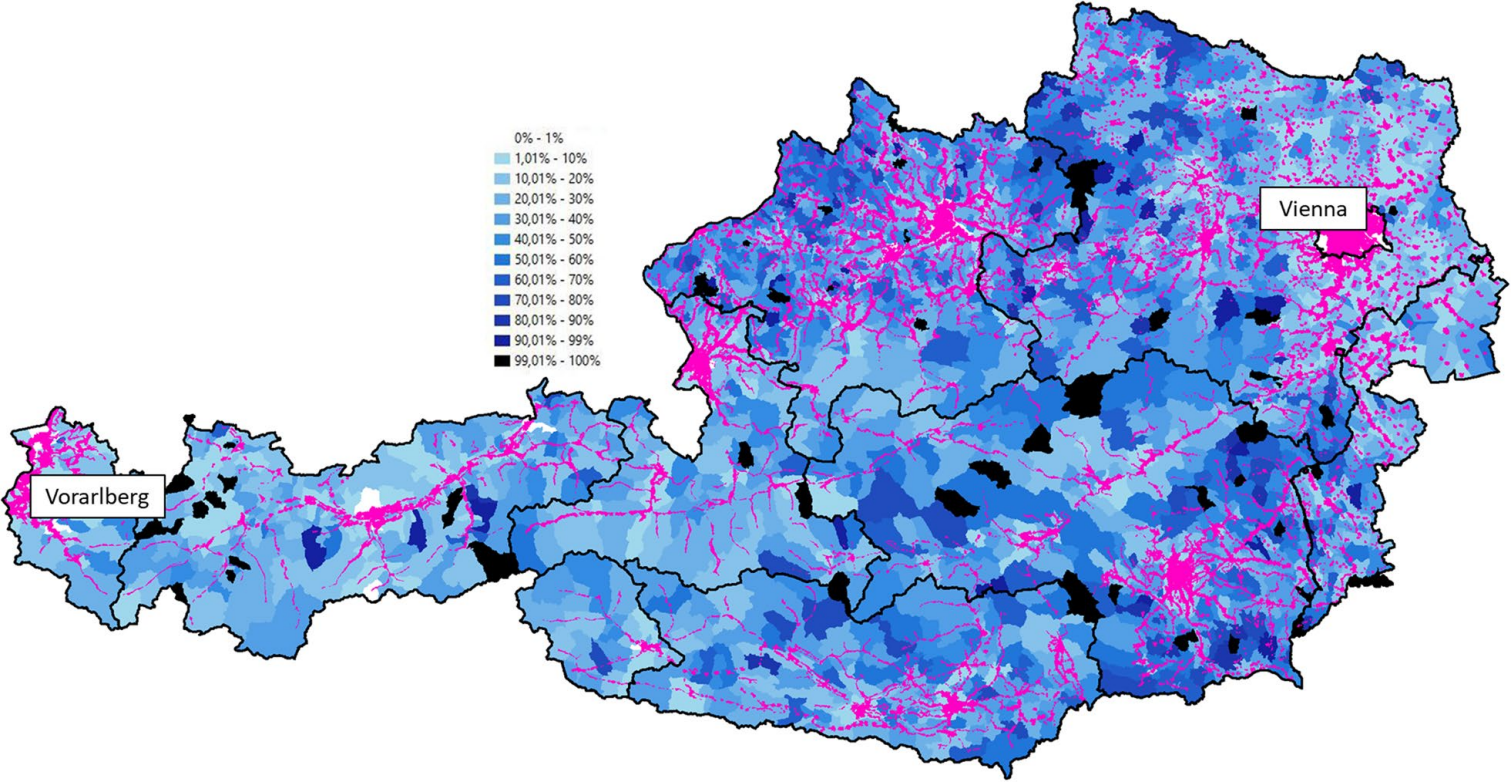
Public transport (PT) coverage of inhabitants in Austria according to PTSQL, on a typical weekday. Pink areas are covered by SQL classes A-G, blue shaded areas show the share of inhabitants on municipality level that are not covered with PT according to PTSQL classes [6]

Combining PTSQL with geoencoded population data, the analysis shows that on weekdays,15.4% of inhabitants have no access to PT according to PTSQL, and on weekdays during school holidays, 20.0% have no access to PT, corresponding to 1.78 million people. At workplaces, 10.4% of employees do not have basic PT access on weekdays and 13.5% on weekdays during school holidays. In general, cities and regional centres show higher classes and more PT coverage than rural areas. The geographic distribution of PT coverage is shown in Fig. 1. The pink areas show that larger cities and valleys in mountainous regions are already covered well by PT services while the blue areas show the share of inhabitants without access to PT. Darker shades represent a higher share of inhabitants without access to PT. Although these areas cover for the majority rural regions, there are some exceptions. On the one hand, there are rural areas with relatively high levels of coverage by PT, for example in Vorarlberg, the most western Land. On the other hand, there are less rural areas such as in the surrounding area of the capital city Vienna that show little PTSQL coverage.

## Stakeholder workshops

### Methods and organization

We conducted workshops with professionals from the transport sector as well as with citizens in the role of everyday users of mobility services (user workshops). In both types of workshops, the Holistic Pattern Mining method was applied (approach by Bauer and Szucsich [3], based on Iba and Isaku [21]. Four workshops with professionals and three user workshops with different groups of users were carried out from April to June 2021. Since the workshops took place during the COVID-19 pandemic with according containment measures, they were carried out solely online. We combined a video conference tool and online sticky notes on a digital whiteboard to save all results of the discussion for analysis.

Participants in the workshops with professionals included four women and 18 men, coming from different areas of Austria, including mountainous regions as well as flat areas, large cities, small towns and rural areas. Their occupations included: municipal officers, public transport operators, regional association of municipalities, transport planners, researchers, lobbying organisations and mobility service providers. User workshops included 28 women and 12 men between the ages of 21 and 55, coming from different geographical locations. Their highest education levels differ, from compulsory school to university degree, and occupations were: social workers, researchers, consultants, construction managers, kindergarten teachers and nurses.

The participants were asked two introductory questions before going into an open discussion, guided by a moderator. The questions asked were: *How do you imagine a nationwide “Mobility Service Guarantee”?* and *Which services could/should be included?* In the course of the workshop, more detailed questions were asked by the moderator:

- *Which goal could/should be achieved by introducing an MSG?*
- *Which measures are necessary to make the MSG economically feasible?*
- *Which measures are necessary to achieve high acceptance of users of the MSG?*
- *Which kind of trips, and spatial areas could/should be covered by the MSG?*

Evaluation of the workshop results was done by a structuring qualitative content analysis according to Kuchartz [26].

### Results

Notable differences are observed between the workshops with professionals and the one with everyday users. The professionals focused on the allocation of responsibility and funding for mobility services between federal and regional governments as well as PT agencies. Another important topic for the professionals was the definition of a clear goal for the MSG and its target group. It was debated whether an MSG should serve as a means to render private cars entirely obsolete or just to make sustainable mobility a little more attractive in order to enable lives without second or third cars in households. Regarding tariffs, several experts pleaded for a uniform ticket and tariff system combining all mobility modes that are part of the MSG and pointed out the importance of making the services affordable for all. Furthermore, spatial differences between cities and rural areas were discussed as well as limitations of guaranteeing access to very remote areas.

The user workshop discussions revolved mostly around questions of affordability, complexity of ticketing systems, comfort, safety and accessibility of mobility systems as well as deployment of new technologies, PT coverage outside of urban agglomerations, PT intervals and connections.

## Existing legal framework

**Fig. 2:**
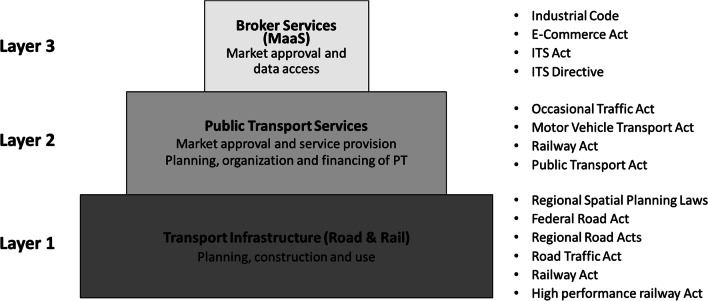
Three legal layers in the Austrian transport sector and corresponding laws and regulations

The analysis of the legal system as well as the legal texts in detail is beyond the scope of transport research, while this is needed for further discussions in the following sections. We provide a brief summary of the current Austrian legal system related to transport and traffic. The complex constructions of different acts in the transport sector in Austria, in particular towards the future regulation of MSG, can be classified into three layers, as shown in Fig. 2.

The bottom layer (Layer 1) is about the legal acts for planning, construction and use of transport infrastructure. It covers both roads and railways but mostly in a separate manner for the two modes. In the context of MSG, infrastructures for pedestrians and cyclists, as well as for railways, are highly relevant. The spatial planning laws (regional acts different in each *Land*) are also highly relevant in that they serve as a basic guidance for transport planning. Currently, the regulations on infrastructure only contain general objectives with regard to guaranteeing a certain transport infrastructure (i.e., its existence and condition). It is not stipulated concretely how to achieve these objectives or what legal consequences are associated with their non-fulfilment. The focus of national and regional regulations is on the service quality for private motor vehicles. In some regional acts, consideration is also given to sustainable transport, such as the Road Act of the Land of Vorarlberg mentioning public transport, cycling and walking.

The second layer regulates the organization and provision of passenger transport services. This includes both private and public services on the road and railway. It covers the regulation of both regular transport services and DRT. All acts on this layer are federal laws that create the prerequisites for transport services of general interest in the form of public (i.e., non-commercial) transport services, which are largely financed by the national and regional governments. This is liaised with the so-called PSO Regulation (EU Regulation 1370/2007), which stipulates the general framework for such publicly financed public transport services. The exact scope of transport services to be provided as public services is not determined and the obligation to provide a nationwide service is not systematically anchored. However, the legal basis for the services that are actually supplied ensures that they meet certain quality criteria and that they are provided on the basis of uniform tariffs set within the public transport associations.

The third layer is about the regulation of mobility platforms. In the context of MSG, this layer is highly relevant to the information and broker services related to transport. The legal framework of ITS stipulates certain aspects of a guarantee for platforms (accessibility to data) and non-discriminatory (i.e., undistorted) presentation of the data to enable end users to make an objective decision when choosing transport services and routes. However, certain elements that would be relevant with regard to an MSG in the context of MaaS platforms such as provision of real-time data are not or not sufficiently regulated by the current legal framework. Without real-time data, MaaS cannot provide their services sufficiently in an expected manner [14, 18, 30, 42].

## Framework definition and limitation of MSG

### Framework definition

We defined an MSG framework based on the political goal, status quo analysis, stakeholder workshops and legal system. Unlike many of the various existing definitions of mobility guarantees (c.f. Sect. 2), our definition does not focus on one particular aspect such as financial compensations but tries to address the political goal of a holistic approach in a comparable manner to the mobility acts in Berlin and France.

The analysis of current PT coverage demonstrates that a considerable number of people in Austria is not served by public transport at the moment (up to 20%), which shows the need for strong measures on the supply side of traditional PT infrastructure and services. In the Austrian context considering the “nationwide” aspect, particular attention has to be paid to the area outside of major cities, where the level of PT coverage is currently low (c.f. Sect. 3).

There are different expectations among professionals concerning the extent of a nationwide MSG, regarding the general goal of such a guarantee and spatial differences (see Sect. 4). They see the need to differentiate between cities and rural areas and pointed out the limitations of covering very remote areas of the country with such a guarantee. We address this by defining varying parameters for cities and rural areas in different scenarios (see Sect. 7).

Both, professionals and everyday users emphasized the need to consider accessibility, affordability, clarity and simplicity of tariff and booking with regard to the MSG.

Reflecting these findings, we understand the goal of the nationwide MSG is threefold:

1. To ensure a sufficient level of mobility services as a condition for equal participation of the population in public life without owning a car (provision of general interest/social or socio-political dimension)
2. To provide non-discriminatory access to these mobility services for everyday journeys (special consideration of barrier-free access)
3. To create an incentive to switch to sustainable forms of mobility (environmental dimension)

The mobility services under the MSG are to be provided in a safe and reliable way to all at non-discriminatory conditions and affordable prices in a certain minimum quality.

In order to reach these goals, certain foundations should be part of the nationwide MSG that we explain with reference to the three legal layers. Regarding the first layer, a sufficient provision and maintenance of transport infrastructure has to be guaranteed for PT and for active modes (e.g., bike lanes and bicycle parking facilities, as well as footpaths and sidewalks) in a certain level of quality. While the infrastructure for PT forms a foundation for the PT services (see below), the provision of high-quality infrastructure for active modes has twofold relevance to MSG: one is to assure the mobility on foot and with bicycles for door-to-door travels, which strongly contributes to the goal 3 above (environmental dimension), while ensuring goal 1 (social dimension) at the same time. The second relevance is to ensure the access to PT services; this is also strongly related to the goal 3 in that the secure access to PT services incentivises the use of it, and eventually ensure the goals 1 and 2, too.

The second layer (PT services) is where the core part of MSG is anchored in the legal system. The MSG itself will serve as a basis to provide a certain minimum level of PT service throughout the country, available to all persons living or working within defined settlement areas. Several parameters will have to be set such as a maximum distance to PT stops or minimum density of PT stops, frequency of services and operating hours. These parameters may vary depending on regional characteristics and further details have to be researched and discussed in the future. In the description of scenarios below, we provide first suggestions.

In addition to PT services themselves, the possibility of compensation for cancelled services or missed connections, similar to the existing form, may also be incorporated into the MSG and could be extended to DRT and car-pooling services.

Related to the third layer of broker services, the MSG will ensure a framework for the development of open and neutral mobility platforms that provide information and brokerage of transport services in a non-discriminatory and undistorted manner. This means that no particular service provider should be preferred in any of such mobility platforms and all platforms should have access to the information from all service providers. Additionally, the MSG may incorporate platforms providing a way to network private vehicles for car-pooling.

### Limitations

As described above, The MSG primarily covers everyday trips of people who live and/or work in Austria. It applies to the physical mobility of persons, and “virtual” mobility such as video-conferencing and mobility of goods (e.g., grocery deliveries) are out of the scope. Occasional trips (e.g., tourism) and virtual mobility as well as freight transport share many aspects with everyday mobility and could be beneficial for the sustainability goals; however, within the scope of this research, which serves as the first gedankenexperiment (thought experiment) towards MSG, we limited the scope to everyday mobility of persons so that the essential goals of MSG are addressed in the most direct manner.

In light of the social and sustainability goals (goals 1 and 3), private motorized transport is excluded from the MSG. The MSG considers only PT (both regular ones and DRT), as well as cycling and walking, mainly as a feeder mode for PT. Nevertheless, the guarantee may also apply to car-pooling services, in order to help increase vehicle occupancy rates of private cars as a kind of “bridging service” during the transition period. As it will take a long time to implement measures to realize the defined service level fulfilling MSG, it is reasonable to tap into the potential of reducing car trips through pooling in the earlier transition period until the final service level of an MSG will have been achieved nationwide. Nevertheless, a potential rebound effect at later stages of transition to full service under MSG must be taken into consideration in that pooling services might lead to more attractive car trips than available PT.

In our proposed definition, the MSG may cover the provision of mobility platforms for carpooling, but not the ones for vehicle sharing as well as not the provision of vehicle sharing services per se. With the currently known forms of vehicle sharing services (e.g., station-based and free-floating services), it is not possible to guarantee the availability of vehicles in the outside of densely populated areas throughout the country. In addition, vehicle sharing calling for driver’s license (e.g., car sharing) cannot be used by those who do not possess it. Such services would exclude children, elderly people and others who are not capable or allowed to drive by themselves and therefore do not fulfil the aforementioned non-discriminatory goal of MSG (goal 2). Furthermore, in light of the sustainability goal (goal 3), free-floating vehicle sharing services are known to encourage the use of private motorized modes [4, 28]. Thus, vehicle sharing is out of the scope of MSG, but it is still a welcome addition to MSG.

There are further aspects that are important to consider in relation to MSG but beyond the scope of this study. This includes the comfort and added-value services of PT (e.g., ambient design, air conditioning on board) as well as disruption management. Another important issue is the cohesion between MSG and spatial planning. Travel needs derive from the dispersion of uses in space. Therefore, a supporting strategy for minimizing travel needs could be to provide more mixed use areas with services such as grocery stores and doctors in rural areas, in contrast to providing mobility services to reach such uses further away. Those aspects will have to be expanded upon in further research on the topic.

## Scenarios of MSG

Based on the framework definition in Sect. 6, we defined five design scenarios towards nationwide MSG in the Austrian context. In this section, we discuss relevant transport-planning parameters and possible ranges for the different scenarios. Table 1 and Table 2 show the five design scenarios and one scenario without MSG for comparison. Scenario 0 “No MSG” shows the current parameters without MSG. Scenario 1 and 5 are to be understood as the plausible lower and upper limits, with the first as the most basic version of an MSG, and the latter as a “Questionable utopia” where PT is available all day long, every day for everyone. Scenario 2 and 3 are intermediate ones with the first one focusing on active mobility, especially cycling as a feeder mode for PT and the second considering increased car-pooling as a substitute for PT in rural areas. Scenario 4 considers a high degree of PT supply but also extensive push-measures to curb private car use.

**Table 1:** Design scenarios for a nationwide mobility service guarantee – public transport parameters

Scenario	“No MSG”	“All regions aboard”	“Focus active mobility”	“Focus car-pooling “	“Goodbye private car”	“Questionable utopia”
PT quality improvement	No improvement	Only in undersupplied rural areas	Only in undersupplied rural areas	Only in undersupplied rural areas	In all areas (rural and urban)	In all areas (rural and urban)
PT timetable	Different for school days	Same on all weekdays	Same on all weekdays	Same on all weekdays	Same on all weekdays and weekend	Same on all weekdays and weekend
PT spatial coverage	85% PT lines (on school days)	85% PT lines 13% DRT (total 98% inhab.; 100% ASA)	85% PT lines 13% DRT (total 98% inhab.; 100% ASA)	85% PT lines 6% DRT (total 91% inhab.; 94% ASA)	98% PT lines 2% DRT (100% inhab.)	98% PT lines 2% DRT (100% inhab.)
PT operating hours (guarantee)	(No guarantee)	6 a.m.–8 p.m	06 a.m.–10 p.m.	6 a.m.–10 p.m	6 a.m.–11 p.m.	24/7
Peak hours	–	6 a.m.–9 a.m. & 3 p.m.–6 p.m	6 a.m.–6 p.m	6 a.m.–6 p.m	6 a.m.–6 p.m	6 a.m.–8 p.m.
*Urban area*^1^						
Max. distance to bus	–	300 m				
Max. distance to train	–	1,250 m				
Min. interva^2^	–	15 min/30 min			10 min/30 min	
*Rural area*						
Max. distance to bus	–	500 m				300 m
Max. distance to train	–	1,250 m			1,250 m	
Max. distance to DRT^1^	–	300 m	300 m/door-to-door		Door-to-door	
Min. interval^1^	–	210 min		120 min/210 min	60 min/120 min	30 min/60 min
PT tariff	22% seasonal ticket	22% seasonal ticket		50% seasonal ticket	70% seasonal ticket	Free PT

^1^Classification according to urban-rural-typology of Statistik Austria https://www.statistik.at/wcm/idc/idcplg?IdcService=GET_PDF_FILE&dDocName=108332 (Table 1); no DRT in urban areas

^2^Peak hours/off-peak

**Table 2:** Design scenarios for a nationwide mobility service guarantee – active mobility, sharing and push measures

Scenario	“No MSG”	“All regions aboard”	“Focus active mobility”	“Focus car-pooling “	“Goodbye private car”	“Questionable utopia”
Active mobility	No improvement measures	No improvement measures	Strong expansion of infrastructure for walking and cycling	Moderate expansion of infrastructure for walking and cycling	Strong expansion of infrastructure for walking and cycling	
Pooling incentive	No incentive			Incentive for private individual to offer rides	Initial incentive, gradual reduction until 2040	
Tariff car-pooling	Pay-as-you-go (km-dependent price)			Integrated in PT ticket		Free of charge
MaaS platform		Integrated mobile application for PT & DRT^1^	Integrated mobile application for PT & DRT plus bike sharing and parking facilities	Integrated mobile application for PT & DRT plus car-pooling services	Integrated mobile application for PT & DRT plus bike sharing and parking facilities	
Push measures	CO_2_-Price as planned^2^	No additional push measures	Speed limit 30 km/h in built-up areas Traffic calming measures in urban areas	Establishment of HOV lanes (for cars with > 1 person)	Speed limit 30 km/h in built-up areas Traffic calming measures in urban areas Additional road toll No approval of fossil vehicles after 2030	
Sharing		Car sharing as welcome addition, not guaranteed	Support for station based bike sharing year-round, especially in urban areas	Car sharing as welcome addition, not guaranteed	Car sharing as welcome addition, not guaranteed; support for bike sharing	
Spatial planning	No measures	No measures	Focus on densification		Focus on densification, including additional financial incentives	
Transfer hubs	Integrated interval timetable (IIT) for trains from 2025 on				Additionally IIT for buses from 2030 on	

^1^Including routing, ticketing, real time information

^2^Starting in July 2022 with 30 EUR/tCO2; 35 EUR/tCO2 in 2023; 45 EUR/tCO2 in 2024; 55 EUR/tCO2 as from 2023; https://orf.at/stories/3230987/

### Public transport parameters

Table 1 shows parameters for PT. The scenario factor “PT quality improvement” shows where quality of PT is improved, ranging from only undersupplied areas to all urban and rural areas. The timetable definition shows if there are differences in PT timetables on school days, weekdays and weekends/holidays. For the definition of spatial coverage, we draw on the analysis of Brezina and Hammel [6] and differentiate if areas belong to the ASA (adapted settlement area—based on the definition by the national statistics bureau, adapted by the data from OpenStreetMap) and if they are covered by PT in a PTSQL class according to Hiess [20]. The maximum distance to the nearest bus stop for all households and workplaces within ASA is defined between 300 and 500 m and 300 m or door-to-door for DRT services. This is comparable to the suggestion of Gipp et al. [13] (max. 300 m for 80% of inhabitants) and in line with evidence of accepted walking distances [40]. Maximum distance to railway stations is set to 1,250 m in line with the PTSQL definitions and larger catchment area compared to bus stops [20, 40].

Operating times are chosen from 6 a.m. to 8 p.m. in the first scenario, based on the period during weekdays where most of the traveling takes place [11] and the PTSQL definition [20] and up to 24/7 in the utopian scenario Regarding pricing, we consider the availability of a subscription ticket similar to the recently introduced “Climate Ticket”^1^ that costs EUR 1,065 per year for all public transport services in Austria and set a share of PT users owning a seasonal ticket (22%–70%). Other PT users are considered to pay for a single trip with the price depending on distance. In scenario 5, PT and DRT services are free of charge.

### Active mobility, sharing services and push measures

Table 2 shows the parameters for active mobility, sharing services and push measures to curb the use of private cars, which are only described qualitatively. A strong expansion of infrastructure for active mobility should include not only bicycle paths and sidewalks but also bike parking facilities at PT stations. Some of the push measures also act as pull measures for active mobility (e.g., 30 km/h speed limit, traffic calming measures). Regrading car-pooling, there are incentives and tariff integration in the “Focus car-pooling” scenario as well as for “Goodbye private car” and “Questionable utopia”, although the latter ones include a gradual reduction with increasing PT coverage in order to prevent rebound effects of higher car use. Integration of services in a MaaS platform is considered in different levels for the scenarios, although only the regulation and not the provision of such platforms is part of the MSG. Similarly, vehicle sharing services are not part of the MSG but defined in different intensities for the scenarios, since they are seen as a welcome addition and are expected to be supported by the public. The factor transfer hubs indicates implementation of the integrated interval timetable (IIT) [38].

## Conclusion

We developed a framework definition and design scenarios for a nationwide MSG in Austria that are based on the current legal system, political objectives that have been manifested in official strategy documents, the status quo of travel behaviour and PT coverage in Austria, and stakeholder expectations that have been identified in workshops. In line with this, the guarantee has the goals of ensuring a sufficient level of mobility services, providing non-discriminatory access to these services and creating an incentive to switch to sustainable forms of mobility for everyday trips of people who live and/or work in Austria.

We identified a clear distinction between three different legal levels as reference points for the implementation of an MSG. We refer to these layers when presenting the foundations that have to be provided in order to reach the above defined goals. First, physical transport infrastructure has to be provided in a certain quality and quantity that forms the basis for services. Second, public transport services fulfilling minimum criteria have to be provided. Third, a legal framework needs to ensure the development of open and neutral mobility platforms, especially with regard to sharing services and the combination of transport modes.

In order to show different designs with more detailed parameters defining the quality criteria, we propose five scenarios that show the range of possible designs of MSG. They differ in temporal and spatial coverage of the MSG and different quality level of services provided. The definition of those parameters for implementation of the MSG remains to be discussed further.

Compared to existing concepts of mobility guarantees that focus only on single aspects, our approach is more comprehensive while at the same time being more specific than general goals of mobility guarantees such as in the French mobility act (LOM LOI n° 2019–1428). We therefore provide a more tangible definition that can serve as the basis for implementation in the Austrian legal system as well as a template for assessment of MSGs in other countries.

Additionally, the study can serve as a starting point for further analyses such as modelling the impacts of an MSG on the travel behaviour and its possible contribution to reaching sustainability goals.

We conclude that the MSG could represent an important element of sustainable transport policy, although many questions remain unanswered. Future research should, on the one side, look into the practical implementation in terms of PT supply in rural areas. Even though there are ongoing pilot project in this regard, the organization and financing of rural PT still presents a particular challenge in the Austrian context. On the other hand, further research should consider the impacts and possible rebound effects due to the introduction of the MSG.

## Data Availability

The German-language working paper containing the full data for Sects. 2 and 3 are made available upon request.

## Abbreviations

COVID-19: Coronavirus disease 2019
DRT: Demand-responsive transit
EU: European Union
IIT: Integrated interval timetable
ITS: Intelligent transport systems
MaaS: Mobility as a service
MSG: Mobility service guarantee
PT: Public transport
PTSQL: Public transport service quality level

## Footnotes

1 https://www.klimaticket.at/
